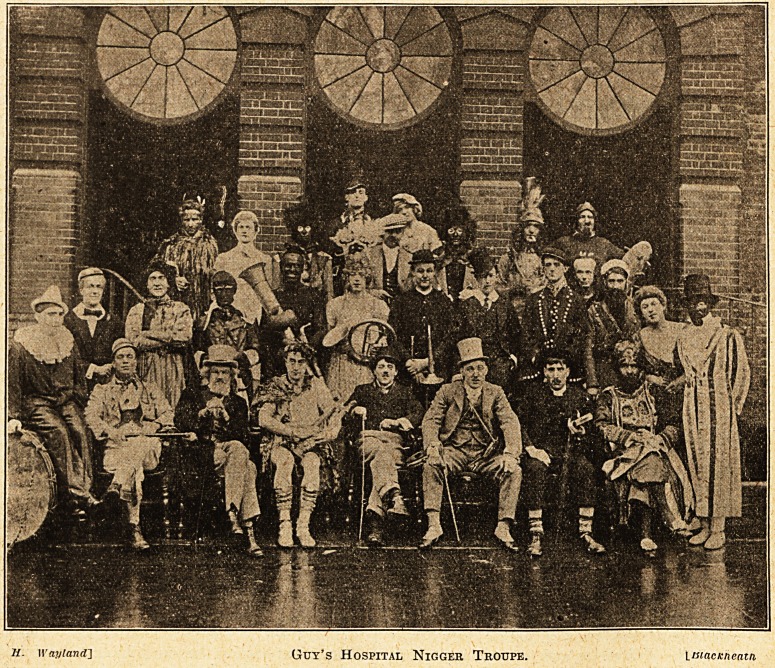# Hospital Music and Christmas Festivities

**Published:** 1916-12-16

**Authors:** 


### HOSPITAL MUSIC AND CHRISTMAS FESTIVITIES.

HOSPITAL music is something which the heathen, who have probably never heard it, may scoff at and deride. Yet those who have lived longest in hospitals and know most about them must be aware, that where there is even one member on the staff who has some knowledge and a keen apprehension of the value of music, there will generally be found a cultivated choir and probably a small, or even a large, orchestra of amateur musicians, who give strength and courage to the patients, and cheer to the convalescents and hospital workers throughout the year. Carol singing is fostered in some hospitals until it may attain, on occasion, a high standard of merit. A sister, who is also a good woman and finds encouragement and hope in music, arranges the singing in her ward at the time of the Angelus, when it arouses and secures the attention of the man in the street. Those who doubt this might seek occasion and ask permission to stand in the quadrangle of the Royal Free Hospital, which is situated off the Gray's Inn Road, at the hour named, when the evening hymn is being sung. We have already called attention to this in THE HOSPITAL, and have given personal experience of its effects. The presence of music in hospitals is not confined to the Metropolis, thank God, but extends throughout the country. Indeed, in those hospitals which are administered with adequate spirit and apprehension, Christmas Day is made memorable, from the manner in which it is kept, and in the keeping of it music has its proper place.

As an act of gratitude and appreciation we have ventured to reproduce the group of musicians, being Guy's Hospital students present and past, whose portraits we are enabled to give in the picture on this page. Last year we gave an account of Christmas Day at Guy's Hospital, and described the wards on that day as possessing a wonderful atmosphere of unselfish effort. No one of all the workers rendered more effective or grateful or prolonged personal service to the patients and the sick, than the members of the Guy's Hospital Nigger Troupe. The illustration contains portraits, and is so worthy of preservation. This is really the first time we have been able to give such an illustration; but our hope is that, through the public spirit and kindness of someone connected with each hospital, where there is a choir, or troupe, or band to lend brightness and

H. Wayland] GUY'S HOSPITAL NIGGER TROUPE. [Blackheath give pleasure at the Christmas festivities, justice will be done to the voluntary workers and to their institution by sending us a photograph, that we may have the satisfaction of reproducing it when opportunity offers.

The writer, as an old Guy's man, knows that thorough is the watchword of all connected with Guy's Hospital, and that any practitioner or nurse, whose Alma Mater it is, has necessarily acquired a knowledge of the value of thoroughness in all things. Certainly the Guy's Hospital Troupe are thorough, untiring, bright, merry, joyous fellows, who warm the heart and brighten the countenance of everyone, who is fortunate enough to see and enjoy their unselfish efforts on successive Christmas Days.

## Figures and Tables

**Figure f1:**